# The Coronary Artery Changes and Complications Caused by Takotsubo Syndrome: A Review With Novel Observations

**DOI:** 10.31083/RCM43182

**Published:** 2025-09-23

**Authors:** Shams Y-Hassan

**Affiliations:** ^1^Department of Cardiology, Karolinska Institute and Karolinska University Hospital, S-141 86 Stockholm, Sweden

**Keywords:** Takotsubo syndrome, coronary thromboembolism, coronary micro-fistula, myocardial infarction, acute coronary syndrome

## Abstract

Takotsubo syndrome (TS) is an acute cardiac disease entity with a clinical presentation identical to that of an acute coronary syndrome (ACS). The terms tsubo- or takotsubo-shaped were introduced at the beginning of the 1990s to describe the silhouette of the left ventricle during systole in patients with a clinical picture similar to myocardial infarction (MI) but with no obstructive coronary arteries. Notably, TS is not a MI and is caused by a different pathogenic mechanism. Innumerable emotional and physical stress factors have been reported to trigger TS, with ACS identified as one of the known physical trigger factors for TS. The myocardial stunning, which is the characteristic feature of left ventricular wall motion abnormality (LVWMA) in TS, was first reported in patients with ACS and also used to describe LVWMA when the term takotsubo-shaped was introduced in 1990. Cases, series of cases, and studies on that ACS may trigger TS have been reported. TS is also known to cause numerous cardiac complications, such as heart failure, pulmonary edema, cardiogenic shock, life-threatening arrhythmias, left ventricular outlet tract obstruction, and cardiac thromboembolic complications. In addition to MI caused by coronary thromboembolic complications, TS may also cause other changes or complications in the coronary artery system, including the intramyocardial resistance microcirculation, the intramyocardial conductance vessels as the septal branches from both the left anterior descending artery and the posterior descending artery, the coronary segments with myocardial bridging, the epicardial coronary arteries, and coronary artery–left ventricular micro-fistulae (CALVMF). This paper reviews these coronary artery complications or changes caused by TS. Notably, the transient compression of CALVMF during the acute stage of TS and reappearance during normalization of the left ventricular function represent novel observations.

## 1. Introduction

Takotsubo syndrome (TS) is an acute cardiac disease entity with a clinical picture resembling that of an acute coronary syndrome (ACS) [1, 2]. TS is not an ACS or acute myocardial infarction (MI), and most of the patients with TS have normal coronary arteries [3, 4]. However, chronic obstructive coronary artery disease and TS may coexist [5, 6]. Moreover, ACS is one of the physical trigger factors for TS [7, 8, 9, 10, 11, 12]. Myocardial stunning represents the characteristic feature of the left ventricular wall motion abnormality (LVWMA) in TS, and was first described in patients with ACS [13]. Sato *et al*. [1] and Dote *et al*. [2], who introduced the term tsubo- or takotsubo-shaped, also described the LVWMA in their patients as post-ischemic myocardial stunning. Indeed, ACS triggering TS has been reported in many cases, series of cases, and studies [7, 8, 14, 15]. Conversely, TS may be complicated by coronary artery thromboembolic occlusion [16] and coronary artery complications or changes involving the intramyocardial resistance microcirculation [17], the intramyocardial conductance vessels as the septal branches, coronary segments with myocardial bridging [18], epicardial coronary arteries, especially the left anterior descending artery (LAD) and posterior descending artery (PDA), and the coronary artery–left ventricular micro-fistulae (CALVMF); this last observation represents a novel finding. This paper reviews the different coronary artery changes and complications caused by TS.

## 2. Takotsubo-Mediated Coronary Artery Complications and Changes

Two mechanisms are known to cause coronary artery complications or changes in TS. The first mechanism is through the development of left ventricular thrombus (LVT), especially in the mid-apical pattern of TS, through associated coronary thromboembolic complications [16]. The second is a mechanical one, which is the compression of both the resistance intramyocardial microcirculation [17] and the conductance macrocirculation (intramyocardial and epicardial) caused by myocardial stunning in TS [18].

### 2.1 Coronary Thromboembolic Complications in Takotsubo Syndrome

LVT formation and cardioembolic events are among the serious complications associated with TS [19, 20, 21, 22, 23], in addition to further cardiac complications, such as heart failure, pulmonary edema, cardiogenic shock, life-threatening arrhythmias, left ventricular outlet tract obstruction, mitral regurgitation, cardiac rupture, and death [5, 24]. LVT has been reported in 1–8% of patients with TS [5, 16, 20, 21]. Thromboembolism has generally been reported in 2–14% of patients with TS [16, 19, 21, 22, 23, 25]. Cardioembolic events have occurred in 17–33% of those patients with LVT in TS [16, 20, 21, 25]. Cases of cardioembolic events in the absence of detectable LVT have also been reported [22, 26, 27]. The LVT occurs mostly in the apical or mid-apical patterns of TS, and at the apical region in the left ventricle, where slow blood flow is a contributing factor for LVT formation [20, 25]. LVT has also been reported (but rarely) in apical sparing patterns in TS [21, 27], where the thrombus may occur adjacent to the papillary cardiac muscles [21].

**Fig. 1.**
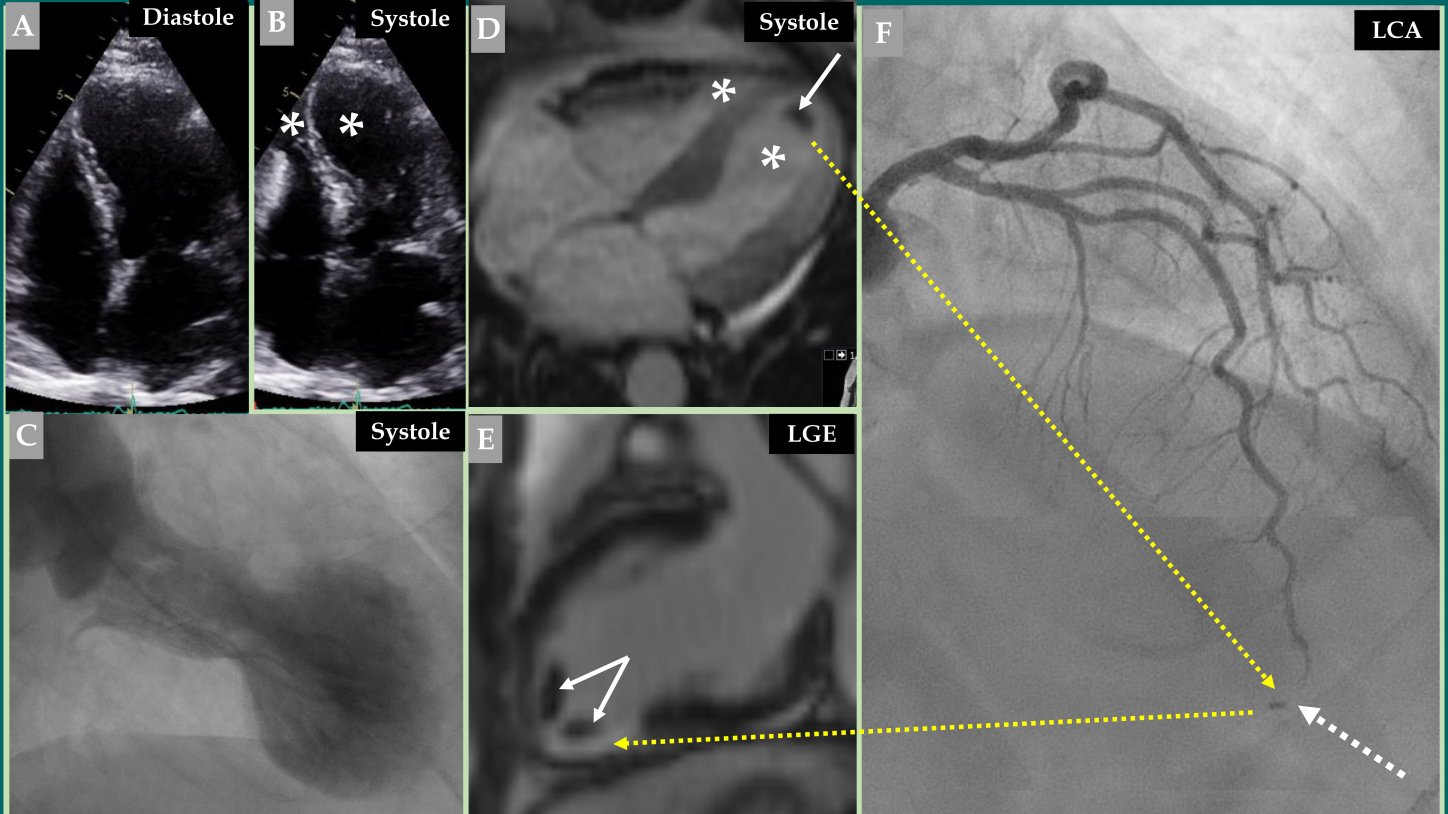
**Demonstration of a coronary thromboembolic complication in a patient with a biventricular mid-apical pattern of Takotsubo syndrome (TS)**. Echocardiography (A, diastole and B, systole) image of a biventricular mid-apical pattern of TS (B, white asterisk in both left and right ventricles). Contrast left ventriculography (C) of the typical mid-apical ballooning pattern of TS during systole. Cine view of cardiac magnetic resonance (CMR) imaging (D) during systole also reveals a biventricular pattern of TS (white asterisk in both left and right ventricles). This was complicated by left ventricular thrombus (white arrow). Late gadolinium enhancement (LGE) view of the CMR imaging (E) shows two thrombi at the apical region of the left ventricle (two white arrows). This view also shows LGE at the apical-inferior region (broken yellow arrow) corresponding to the occluded apical segment of the left anterior descending artery (LAD) (F, broken white arrow). Contrast left coronary artery (LCA) angiography in the right cranial anterior oblique projection shows atheromatous changes with occlusion of the apical segment of LAD (F, broken white arrow) caused by embolization from the left ventricular thrombus (F, broken yellow arrow).

The most common reported sites of cardioembolic complications are cerebral, renal, and peripheral limb arteries [16, 20, 21, 23, 27]. Embolization to the coronary arteries has also been reported [16, 28, 29]. Notably, TS and coronary occlusion may coexist in some cases, which causes difficulties in determining whether the coronary occlusion is the trigger or the consequence. Y-Hassan *et al*. [16] reported on a 67-year-old woman with a mid-apical pattern of TS triggered by an intense emotional stress. This case was complicated by left ventricular thrombus with an embolic complication to the apical segment of LAD, causing a limited myocardial infarction in the corresponding segment. The small apical LAD occlusion could not explain the extensive LVWMA observed in the mid-apical region caused by TS. That case was also complicated by middle cerebral artery embolization [16]. Fig. 1 presents an illustrative case of a biventricular mid-apical pattern of TS that is complicated by left ventricular thrombi, which have resulted in the LAD embolization at the apical segment, causing acute MI at the corresponding left ventricular region.

### 2.2 Myocardial Stunning-Induced Compression of the Coronary Arteries and Circulations in TS

Myocardial stunning forms the characteristic feature of the LVWMA in TS, where the myocardium in the affected segments is in a state of cardiac cramp. The contractility pattern of the left ventricle during diastole and systole with a rigid akinetic segment in the affected regions and the hyperkinetic segments in the normally contracting regions, causing a valve-like motion of the basal area in the mid-apical pattern of TS and the slingshot-like motion of the apical segments in the apical sparing patterns of TS are important findings that support the state of myocardial cramp in myocardial stunning [30]. The second important finding is the myocardial histopathological findings of contraction bands and hypercontracted sarcomeres seen in patients with TS [31]. This state of myocardial cramp forms the main explanation for the transient compression of intramyocardial microcirculation and macrocirculation, and the epicardial coronary arteries.

#### 2.2.1 Compression of the Intramyocardial Resistance Coronary Microcirculation 

Signs of coronary microvascular dysfunction (CMVD) have been reported in some but not all patients with TS. Sufficient evidence argues for the fact that CMVD represents a secondary process or complication and resolves with the resolution of the left ventricular function [17]. Invasive coronary angiography (CAG) revealed normal coronary arteries in most of the patients with TS [3, 4]. However, it is well-known that invasive CAG cannot visualize the coronary microcirculation (pre-arterioles and arterioles of less than 500 µm), which is one of the limitations of invasive CAG. Different invasive and non-invasive examinations have been used as substitute techniques to study the coronary microcirculation. Some studies using invasive techniques derived from CAG have shown coronary slow flow (CSF), and a significant increase in thrombolysis in MI (TIMI) frame-count (TFC) in patients with TS compared to control subjects [32, 33, 34]. The CSF and a significant increase in TFC in TS are used as signs of microvascular dysfunction. Recently, Stępień *et al*. [35] conducted a retrospective study of 82 patients with TS and identified CSF in 33 (40.2%) of the patients. All 33 patients with CSF-TS had slow-flow in the LAD; 7 (21.2%) had isolated CSF only in the LAD. Moreover, 26 (78.8%) revealed CSF also in the left circumflex artery (LCx) and 11 (33.3%) in the right coronary artery (RCA). This implies that 9% of TS patients had CSF in all three vessels (LAD, LCx, and RCA), 12.3% had CSF in 2 vessels (LAD and LCx), and 5.74% had CSF in only LAD.

The index of microcirculatory resistance (IMR) and hyperemic microvascular resistance (HMR) represent another important invasive technique used to study the coronary microcirculation [36, 37, 38]. IMR has been measured in the LAD in case reports in patients with TS and was remarkably high, indicating microvascular dysfunction [39, 40, 41, 42]. Recently, Ekenbäck *et al*. [42] evaluated 27 patients with TS for CMVD and compared them with patients with ischemia and non-obstructive coronary arteries (INOCAs). The investigators found a higher incidence of CMVD in TS patients compared to INOCA patients (78% vs. 44%; *p* = 0.01) with significantly higher IMR. Other invasive and noninvasive techniques have been used and showed that a significant number of patients with TS have signs of CMVD and have been reviewed elsewhere [17, 43].

Nevertheless, some studies have shown signs of normal microvascular function in patients with TS [44, 45]. Furthermore, patients who had shown signs of CMVD did not exhibit CMVD in all three coronary artery distributions. Some patients had CMVD in one or two vessel distributions [33, 46], as demonstrated by Stępień *et al*. [35]. CMVD has been reported to be more prevalent and more pronounced in the LAD distribution [47, 48, 49]. Montone *et al*. [50] studied 101 patients with TS and found CSF in only 18 of 101 (17.8%) patients. Most of these patients with CSF, 17 of 18 patients (94%), had CSF only in the LAD. Only one patient had CSF in both the LAD and LCx. Furthermore, patients with CSF had worse myocardial perfusion with significantly reduced myocardial blush grade and quantitative myocardial blush score in the LAD territory compared with patients with normal coronary flow.

Patients who have been investigated for CMVD during follow-up of TS have shown improvement or normalization of microvascular function [33, 39, 51, 52, 53]. Other investigators have observed that the LVWMA improved in parallel with a dynamic improvement in the microcirculation [51, 52, 53]. In a study of 14 patients with TS, Rivero *et al*. [54] found that the mean coronary flow reserve (CFR) was 1.4 and the mean IMR was 53, indicating microvascular dysfunction. The interesting observation was a significant negative linear correlation between the extent of microvascular dysfunction and the time from symptom onset to the IMR measurement, indicating an improvement in the CMVD with time after the acute onset of TS. The findings of these studies suggest that CMVD in TS or the compression of the intramyocardial microcirculation is transient and normalizes following the resolution of the left ventricular dysfunction.

Consequently, signs of CMVD occur in a limited number of patients with TS and may appear in one-, two-, or three-vessel distribution. CMVD is more prevalent and pronounced in the LAD distribution. Moreover, CMVD may be associated with perfusion defects and even with more complications. Meanwhile, CMVD is reversible and normalizes with the normalization of the left ventricular dysfunction. All these findings indicate that CMVD is a secondary process caused by compression of the resistance microcirculation by the myocardial stunning in TS; further support for this mechanism is presented below. However, it should be mentioned that the role of CMVD in the pathogenesis of TS as a cause, consequence, or both remains under discussion. Some investigators have discussed that sex hormonal variation and its effect on endothelial function can predispose to the development of TS. Others believe that the enhanced activity of the sympathetic nervous system and catecholamines play a crucial role in the pathogenesis of TS. This has been discussed in detail elsewhere [17, 55].

#### 2.2.2 Compression of the Conductance Intramyocardial Coronary Arteries (Macrocirculation)

In addition to the intramyocardial resistance vessels (microcirculation), there are also intramyocardial conductance vessels (macrocirculation), such as the septal branches from the LAD and PDA, and the coronary artery segments with myocardial bridging, especially the LAD [56]. The compression of the mentioned intramyocardial conductance vessel may explain the more prevalent and more pronounced signs of CMVD in the LAD system during the acute and subacute stages of TS compared to other coronary arteries. Compression of the septal branches originated from the LAD; meanwhile, the coronary segments with myocardial bridging have also been reported [17, 18, 30, 43].

Systo-diastolic compression of the LAD segments with myocardial bridging during the subacute stage of TS has been described [18]. The relief of systo-diastolic compression has also been documented during the resolution of left ventricular dysfunction [18]. Compression of the septal branches and even the occlusion of the septal branches during systole have been reported in patients with TS [56]. Migliore *et al*. [56] observed transient systolic occlusion of septal branches arising from the LAD segment with myocardial bridging in 4 (9%) out of 42 patients with TS. The compression of intramyocardial septal branches during the acute stage of TS and the release of compression during recovery of left ventricular function argue strongly for the fact that the intramyocardial microcirculation is also compressed during the acute stage of TS and relieved during recovery of the myocardial stunning, explaining the reversibility of CMVD as previously mentioned [39, 54, 57]. Y-Hassan S [18] reported on a 76-year-old woman who presented with a clinical picture of unstable angina pectoris, which triggered a typical mid-apical pattern of TS. Cardiac magnetic resonance (CMR) imaging did not show any late gadolinium enhancement. The patient had coronary stenosis in all the three coronary arteries, including very tight proximal LAD stenosis. One important feature observed during the index presentation was the systo-diastolic compression of a long segment in the middle part of the LAD corresponding to the region of mid-apical ballooning of the left ventricle. The septal branches from the distal segments of the LAD were not visible. These changes were also seen 5 days after the index presentation, directly after the coronary angioplasty of the proximal LAD stenosis. This segment of the LAD showed myocardial bridging with intravascular ultrasound imaging. There was complete normalization of both the LAD segment with myocardial bridging and its septal branches after 26 days, when the left ventricular dysfunction was completely normalized; only mild systolic compression of the LAD was observed during systole. This report represents a case where ACS triggered TS, and the myocardial stunning of TS caused transient systo-diastolic compression of both the LAD segment with myocardial bridging and its septal branches, a typical case where the coronary arteries and the myocardial stunning in TS were really in a state of “civil war”. The findings in that case support the hypothesis that the stunned myocardium during the acute and sub-acute stages of the disease was in a cramp state, causing incessant (during systole and diastole) compression of a segment of the LAD with myocardial bridging. However, it should be acknowledged that the evidence mentioned above is based on case reports, case series, and small studies.

#### 2.2.3 Compression of Epicardial Coronary Arteries by the Myocardial Stunning in TS

**Fig. 2.**
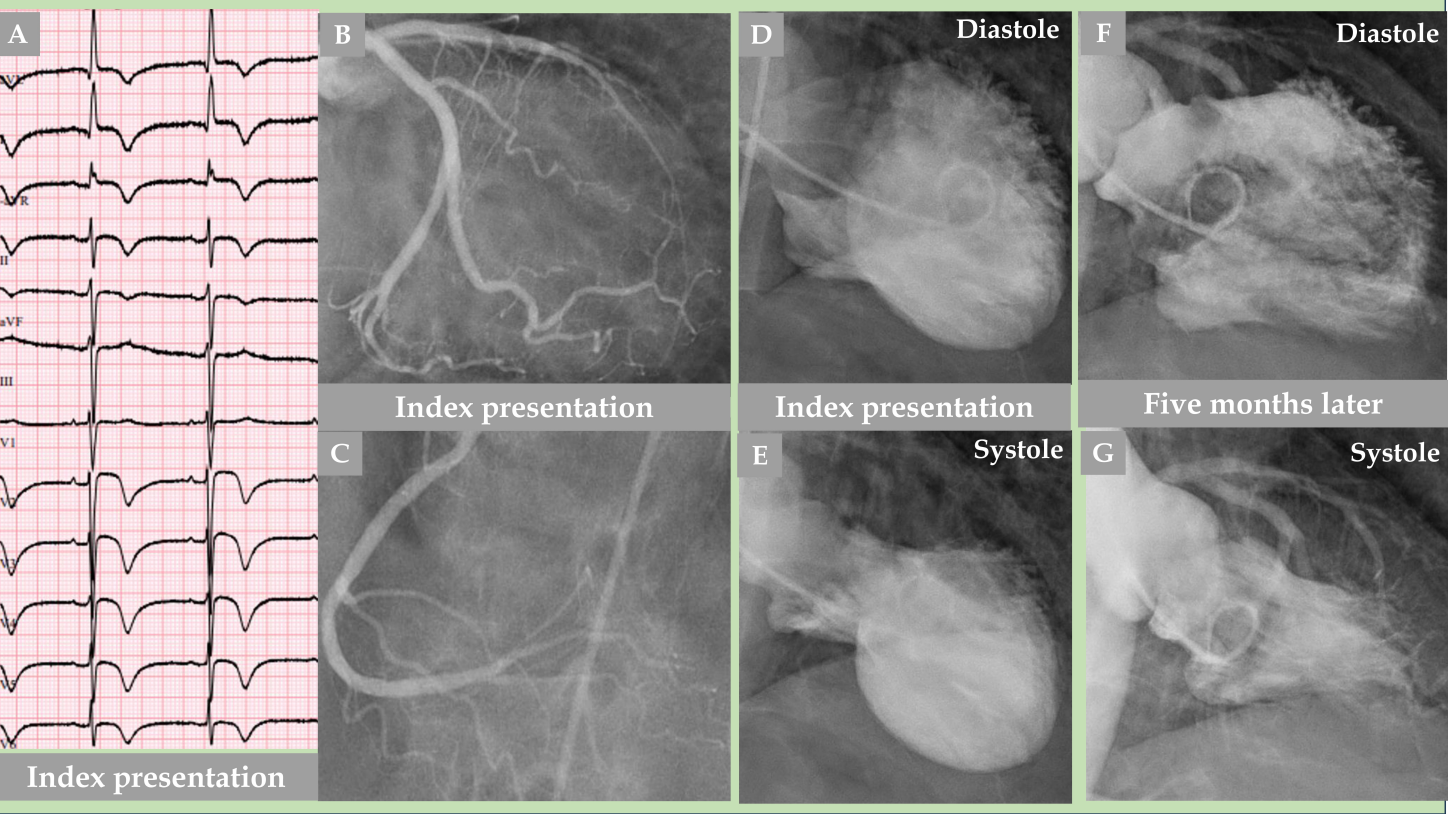
**Demonstration of an older woman with a mid-apical pattern of TS with complete normalization of the left ventricular function after 5 months**. The electrocardiogram (A) shows extensive repolarization changes with certain ST elevations and widespread T-wave inversions. LCA angiography (B) and right coronary artery (RCA) angiography (C) showed no coronary artery occlusion; however, certain other LCA changes are detailed in Fig. 3. Contrast left ventriculography (D, diastole and E, systole) of a mid-apical ballooning pattern of TS. The follow-up contrast left ventriculography 5 months after the index presentation revealed complete normalization of the left ventricular function (F, diastole and G, systole).

**Fig. 3.**
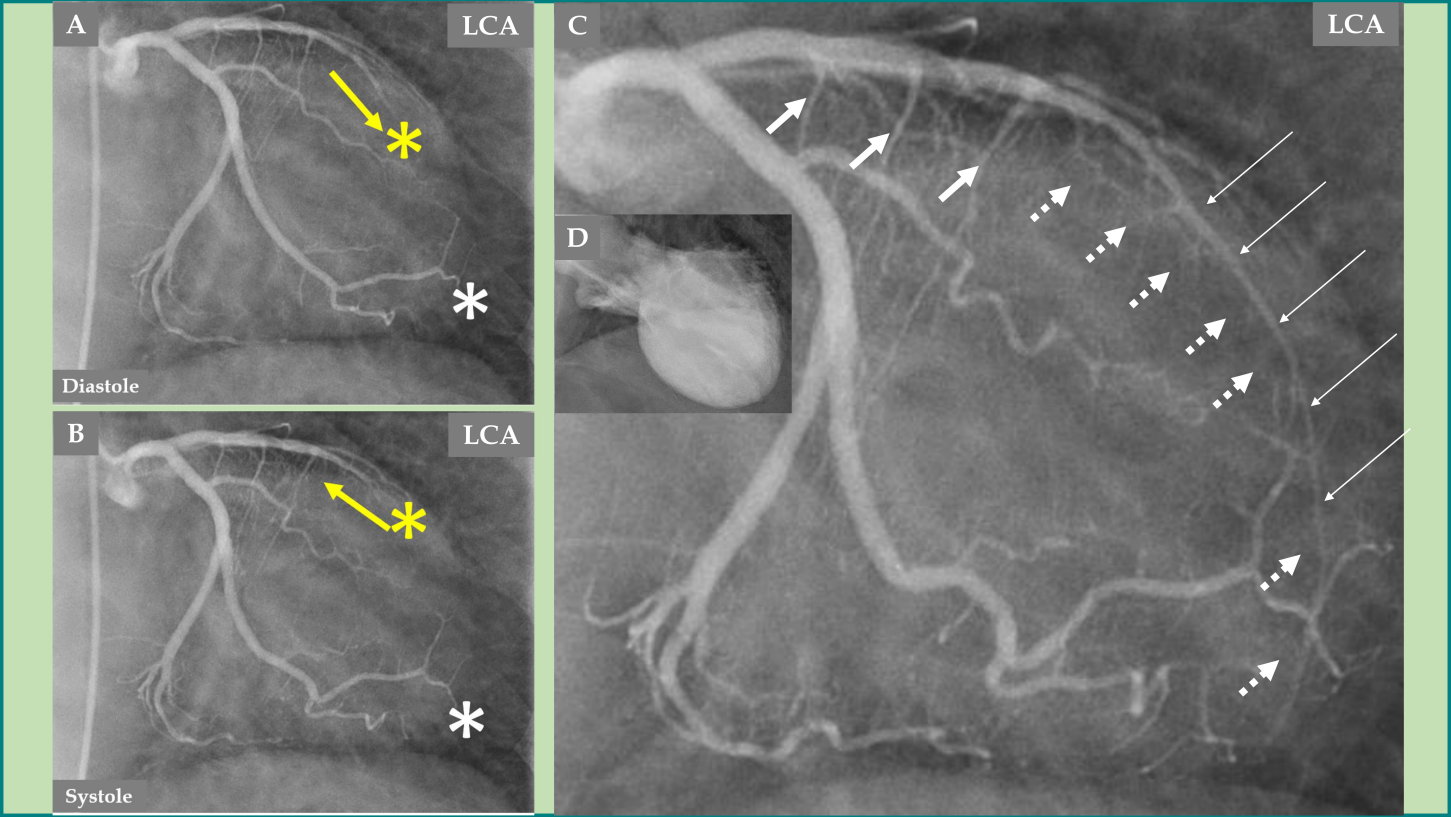
**Demonstration of the LCA changes and complications during the acute stage of TS—the same case as presented in Fig. 2**. The LCA angiography during diastole (A) and systole (B) is clearly seen, where forward flow in the LAD during diastole (A, yellow arrow) and backward flow in the LAD during systole (B, yellow arrow) due to LAD compression during systole. Signs of slow flow in the LAD are also seen compared to an almost normal flow in the left circumflex artery (LCx) (A and B; LAD, yellow asterisk; LCx, white asterisk). The LAD in the distal two-thirds is thin (C, thin white arrows), which illustrates compression by the myocardial stunning caused by TS in the mid-apical region of the left ventricle. The well-filled three septal branches from the proximal segment in the LAD are clearly seen (C, thicker white arrows) corresponding to the normally contracting basal region in the left ventricle, while the septal branches from the distal two-thirds of the LAD are invisible (C, broken white arrows) due to compression by the myocardial stunning in the mid-apical region of the left ventricle. The mid-apical ballooning pattern of the left ventricle in the acute stage of TS is seen in (D).

**Fig. 4.**
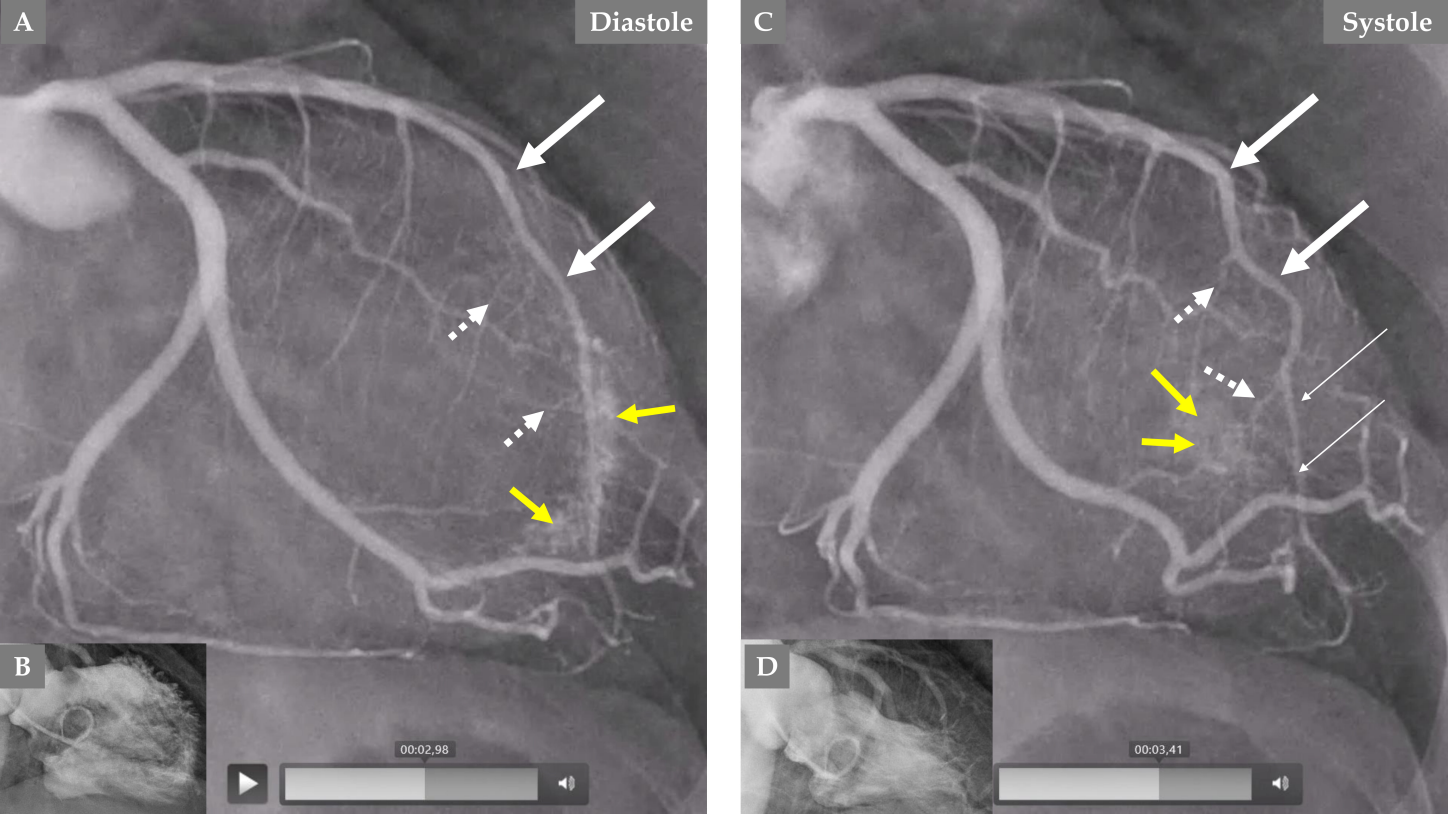
**Demonstration of remarkable changes in the LCA 5 months after the index presentation when the left ventricular dysfunction is completely normalized**. LCA angiography during diastole (A). Contrast left ventriculography during diastole (B). LCA angiography during systole (C). Contrast left ventriculography during systole (D), which shows complete normalization of the left ventricular function. The LAD has a normal diameter, especially in the proximal two-thirds (A and C, thick white arrows), but mild systolic compression in the distal segment (C, thin white arrows). The septal branches from the distal half of the LAD are seen now during both diastole and systole (A and C, broken white arrows). The most remarkable change is the emergence of contrast in the left ventricle (yellow arrows during diastole in A and systole in C) due to reopening of the coronary artery–left ventricular micro-fistulae draining into the left ventricle.

**Fig. 5.**
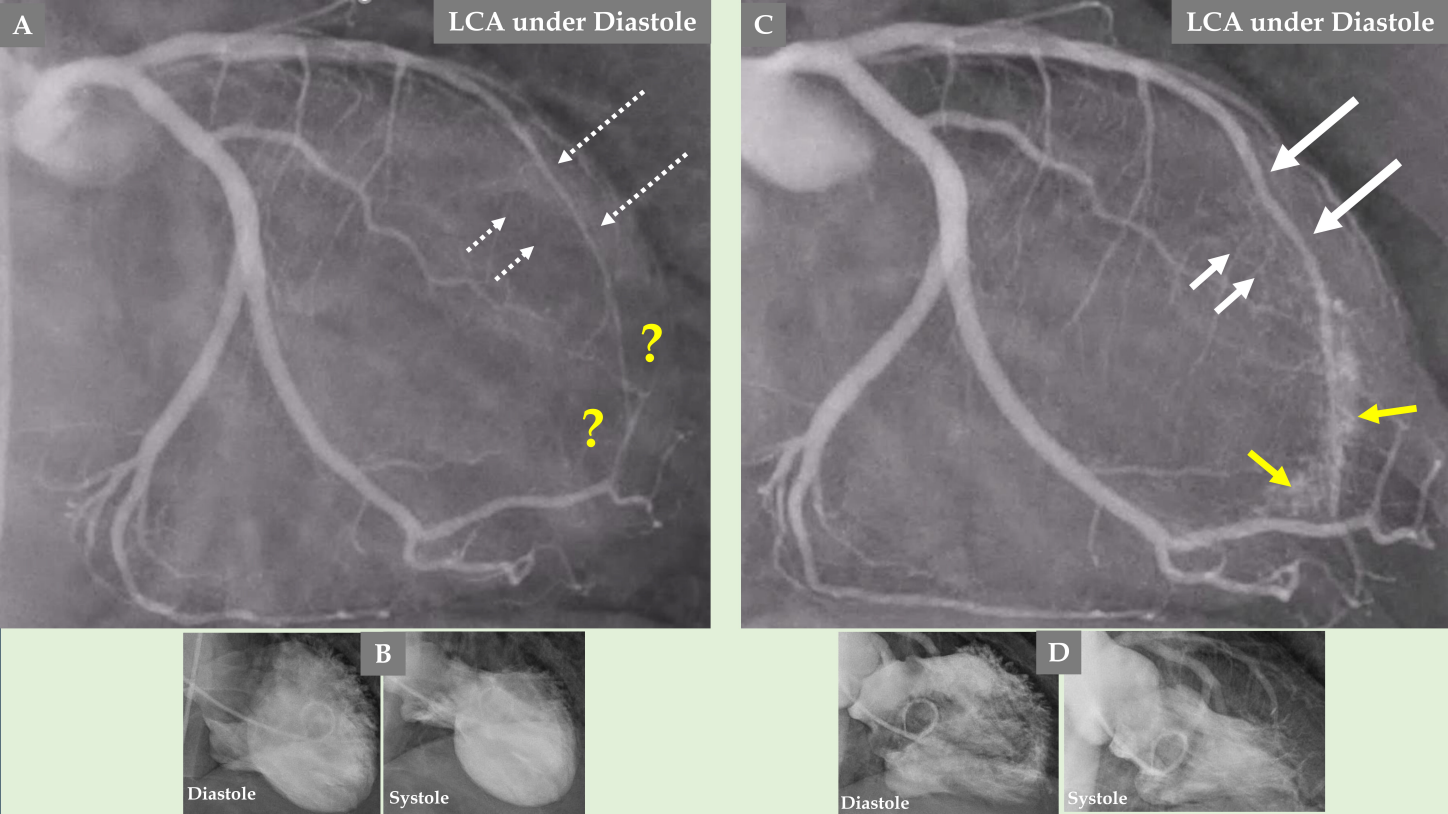
**Comparison of the LCA angiography during the index presentation and follow-up after 5 months**. LCA angiography during diastole (A). Contrast left ventriculography during diastole and systole (B). Both (A and B) are during index presentation, where the patient had a clear mid-apical ballooning pattern of TS. LCA angiography during diastole (C). Contrast left ventriculography during diastole and systole (D). Both (C and D) are during the follow-up, 5 months after the index presentation, where there was complete normalization of left ventricular function. The LAD has a normal diameter in the proximal segment and normal three septal branches in both (A and C); meanwhile, the distal two-thirds of the LAD is thin and compressed (A, broken thin long white arrows), and the LAD is almost normal (C, thick long white arrows) during follow-up. The septal branches from the distal two-thirds of the LAD are practically invisible (A, short broken white arrows). The septal branches from the distal two-thirds of the LAD (C, short white arrows) are clearly seen. There are no signs of coronary artery-left ventricular micro-fistulae (CALVMF) during the index presentation (A, yellow question marks). Clear signs of CALVMF are observed around the distal segment of the LAD in the projection; however, CALVMF are most probably from the distal marginal branch (C, yellow arrows) when all projections of LCA angiography have been analyzed. This figure clearly shows that the LAD, the septal branches, and CALVMF were compressed by the myocardial stunning during the index presentation and were relieved during follow-up when the left ventricular function is completely normalized.

The myocardial stunning in TS not only compresses the non-visible microcirculation in the myocardium, septal branches, and the coronary segments with myocardial bridging, but also the epicardial coronary arteries, which are more pronounced in the LAD [17, 18, 30, 56], and even in the PDA and CALVMF. These last coronary manifestations in TS represent novel observations, which have not previously been debated. These changes are illustrated in Figs. 2,3,4, and 5, in a patient with a mid-apical pattern of TS, where, in addition to the compressed and almost unobservable septal branches from the LAD overlying the akinetic left ventricular segments, the following manifestations are noticed in the LAD vessel and LAD circulation:

(1) The “to and fro” flow is seen in the proximal segment of the LAD (Fig. 3A,B) at the point where the LAD is located on the hinge point between the normally contracting basal segments in the left ventricle and the akinetic regions in the left ventricle because of increased resistance from the stunned myocardial segments. Slow flow is also observed in the LAD compared to the almost normal flow in the LCx.

(2) In addition to the slow flow in the LAD, systo-diastolic LAD narrowing, which is more pronounced during systole, is observed at the distal two-thirds of the LAD overlying the stunned myocardial segments, which compress the LAD (Fig. 3C).

During the follow-up 5 months later and with normalization of the LVWMA, all the above-mentioned changes in the septal branches, the LAD changes, and flow were normalized. Mild distal systolic LAD compression is seen most likely due to myocardial bridging (Fig. 4C).

#### 2.2.4 Compression of the Coronary Artery–Left Ventricular Micro-Fistulae by Myocardial Stunning in TS

An interesting finding, which forms a novel observation, is that no signs of CALVMF was observed during the index presentation with a mid-apical ballooning pattern of TS (Fig. 3C and Fig. 5A); however, during follow-up coronary angiography when the left ventricular dysfunction has completely normalized, there was clear signs of CALVMF, most probably arising from the distal marginal branches (Fig. 4A,C during diastole and systole and Fig. 5C). It is also clearly seen that the contrast staining moves to the left ventricular cavity during systole (Fig. 4C). These micro-fistulae were not visible during index presentation because of the compression by the myocardial stunning (myocardial cramp) caused by TS. The comparison between the index presentation, where there were no signs of CALVMF, and the follow-up 5 months later, where signs of CALVMF appeared clearly, is demonstrated in Fig. 5.

An association between TS and CALVMF has been reported [58, 59, 60, 61]. Elikowski *et al*. [61] reported on a case and reviewed the clinical data of eight other cases with concomitant TS and coronary artery fistula. Moreover, Elikowski *et al*. [61] found specific triggering factors/predisposing conditions in all nine patients and concluded that the coexistence of TS and coronary artery fistula was coincidental. However, compression of CALVMF in the acute and subacute stages of TS has not been reported. This does not mean that TS with compressed CALVMF has not occurred previously; however, to confirm this phenomenon, a new follow-up coronary angiography is required to establish the reappearance of CALVMF when the left ventricular function is completely normalized, as happened in the current illustrated case. The coexistence of TS and non-compressed CALVMF in the limited reported cases is most probably due to mild myocardial stunning, which was not severe enough to cause complete compression of CALVMF. One may discuss the possibility of the appearance of CALVMF in the current case as a complication of TS, but this is highly unlikely; nonetheless, identifying another reasonable mechanism is difficult. This novel observation of the complete absence of signs of micro-fistulae from the coronary arteries to the left ventricle in the acute stage of TS and reappearance after normalization of the left ventricular function further supports the hypothesis that myocardial stunning in TS is in a state of myocardial cramp and that the CMVD is secondary to the compression of the microcirculation by the myocardial stunning. It should be acknowledged that the reappearance of CALVMF was observed in a single case, and this needs to be confirmed in a prospective imaging-based study in a larger TS cohort. One more recommendation to the researchers in this field is to review the follow-up CAG, if performed in these patients, and to search specifically for the reappearance of CALVMF.

## 3. Conclusion

ACS is one of the reported physical trigger factors for TS. However, TS may cause coronary complications, one of which is LVT and coronary thromboembolic complications. The second important coronary complication or change is caused by myocardial stunning, which may cause transient compression of the intramyocardial resistance microcirculation, causing reversible CMVD in some patients with TS. Myocardial stunning may also cause transient compression of the intramyocardial conductance macrocirculation (septal branches from the LAD and PDA and coronary segments with myocardial bridging) and epicardial coronary arteries, especially the LAD. One novel observation is the transient compression of CALVMF. The last observation further supports the idea that myocardial stunning is in a state of myocardial cramp and that the CMVD is secondary to the compression of the microcirculation by the myocardial stunning.

## Abbreviations

ACS, acute coronary syndrome; CAG, coronary angiography; CALVMF, coronary artery–left ventricular microfistulae; CMVD, coronary microvascular dysfunction; CSF, coronary slow flow; HMR, hyperemic microvascular resistance; IMR, index of microcirculatory resistance; INOCA, ischemia and non-obstructive coronary arteries; LAD, left anterior descending artery; LCx, left circumflex artery; LCA, left coronary artery; LVT, left ventricular thrombus; LVWMA, left ventricular wall motion abnormality; MI, myocardial infarction; PDA, posterior descending artery; RCA, right coronary artery; TS, Takotsubo syndrome; TIMI, thrombolysis in myocardial infarction; TFC, thrombolysis in MI frame-count.
